# *Coxiella burnetii* Strains Elicit Distinct Inflammatory Responses in Human Macrophages

**DOI:** 10.3390/pathogens14111101

**Published:** 2025-10-29

**Authors:** Madhur Sachan, Amanda Dragan, Het Adhvaryu, Daniel E. Voth, Rahul Raghavan

**Affiliations:** 1 Department of Medicine, Cardiovascular Division, Brigham and Women’s Hospital, Harvard Medical School, Boston, MA 02115, USA; 2 Department of Microbiology and Immunology, University of Arkansas for Medical Sciences, Little Rock, AR 72205, USA; 3 Department of Molecular Microbiology and Immunology, The University of Texas at San Antonio, San Antonio, TX 78249, USA

**Keywords:** *Coxiella burnetii*, inflammation, IL-17 signaling, macrophage polarization, single-cell RNA-seq

## Abstract

*Coxiella burnetii*, the causative agent of human Q fever, subverts macrophage antimicrobial functions to establish an intracellular replicative niche. To better understand host–pathogen interactions, we investigated the transcriptional responses of human alveolar macrophages (hAMs) infected with virulent [NMI, G (Q212)], attenuated (NMII), and avirulent (Dugway) strains of *C. burnetii*. RNA sequencing indicated that all strains activated proinflammatory pathways, particularly IL-17 signaling, though the magnitude and nature of the response varied by strain. Infections with NMI, NMII or G (Q212) resulted in differential expression of roughly the same number of genes, while Dugway infection induced a stronger transcriptional response. Dugway and G (Q212) tended to polarize macrophages toward M1-like states, whereas responses to NMI and NMII were variable. Cytokine assays of NMII-infected THP-1 macrophages suggested the activation of IL-17 signaling, but only at later stages of infection, and single-cell RNA sequencing of NMII-infected THP-1 macrophages indicated heterogeneity in host response to infection, with distinct subpopulations exhibiting M1-like and M2-like inflammatory profiles. These findings highlight the complexity of macrophage response to *C. burnetii* and underscore the importance of strain-specific and cell-specific factors in shaping host immunity. Understanding these dynamics may inform the development of targeted therapies for Q fever.

## 1. Introduction

Alveolar macrophages typically provide the first line of defense against invading pathogens [1,2]. They secrete cytokines to eliminate pathogens and to recruit other immune cells as part of the initial acute inflammatory response. One of the proinflammatory cytokines produced by Th17 lymphocytes and other immune cells in the lungs is IL-17, which has a protective role against pulmonary intracellular pathogens such as *Mycobacterium tuberculosis* and *Legionella pneumophila* [3,4]. IL-17 binds to receptors on many cell types, including macrophages. It induces antimicrobial peptide production, chemokine secretion, and promotes macrophage polarization toward the proinflammatory M1 state. These responses enhance pathogen clearance but, in some settings, may also drive pathological inflammation [5].

Unlike most other pathogens, *Coxiella burnetii*, the causative agent of Q fever, subverts macrophage antimicrobial functions to establish a replicative niche termed the *Coxiella*-containing vacuole (CCV) that matures by fusing with lysosomal, autophagic, and secretory vesicles [6,7,8]. Q fever is a worldwide zoonosis with significant public health implications [9]. *C. burnetii* infects mammals (mainly cattle, goats, and sheep) and is typically transmitted to humans by aerosols derived from infected animal birth products [10]. *C. burnetii* has been isolated from a variety of hosts and geographic regions [1,11,12]. The original isolate, *C. burnetii* Nine Mile I (NMI), causes acute Q fever, whereas several others have been linked to chronic Q fever, including G (Q212), which was isolated from the heart valve of a person with endocarditis.

In contrast to strains that are pathogenic to humans, Dugway isolates collected from rodents have attenuated virulence in animal models and are not currently known to cause Q fever in humans [13]. Because clinical and environmental isolates of *C. burnetii* require biosafety level 3 (BSL-3) containment, most laboratories use the BSL-2 strain *C. burnetii* Nine Mile II (NMII) to study *C. burnetii*–host interactions. NMII was derived from NMI through serial laboratory passage in embryonated eggs and has lost several lipopolysaccharide biosynthesis genes, which has attenuated its virulence [14].

In this project, to better understand macrophage response to *C. burnetii*, we investigated the transcriptomes of human alveolar macrophages (hAMs) infected with *C. burnetii* strains. We validated the gene expression data by measuring cytokine production and performing single-cell RNA-sequencing (scRNA-seq) of THP-1 macrophages infected with NMII. Our data indicate that proinflammatory pathways, including IL-17 signaling, are activated in *C. burnetii*-infected macrophages, but the inflammatory response varies depending on the bacterial strain and the macrophage subpopulation.

## 2. Materials and Methods

### 2.1. RNA Sequencing and Pathway Analysis

Primary human alveolar macrophages (hAMs) were harvested by bronchoalveolar lavage from postmortem human lung donors, as previously described [15]. hAMs were infected with *C. burnetii* Nine Mile phase I RSA493 (NMI), Nine Mile phase II RSA439 (NMII), Dugway (5J108-111), or G (Q212) isolates at a multiplicity of infection (MOI) of 25. Infected and uninfected cells were cultured at 37 °C under 5% CO_2_ in Dulbecco’s modified Eagle/F-12 (DMEM/F12) medium (Thermo Fisher, Waltham, MA, USA) containing 10% FBS for 72 h. At this time-point, the growth medium was replaced with 1 mL of TRI reagent (Thermo Fisher), and total RNA was extracted, and DNase treated (Thermo Fisher) as per manufacturer’s instructions. Samples were sequenced using the Illumina NovaSeq 6000 platform at Yale Center for Genome Analysis. Differential gene expression analysis was performed by mapping the sequencing reads to the reference human genome (GRCh38) using CLC Genomics Workbench v6.5 (Qiagen, Germantown, MD, USA) and DESeq2 [16]. Differentially regulated genes were calculated using log2 fold-change ≥ 0.75 and an adjusted *p*-value < 0.05 as cutoffs, as we described previously [17]. Core analysis of the differentially expressed genes to find the enriched pathways was performed using Ingenuity Pathway Analysis (IPA, Qiagen) [18]. For pathway analysis, gene clusters were compared with a standard mammal background database using z-score ≥ 1.5 or ≤−1.5 (*p* < 0.05). Normalized counts from the RNA-seq data were used in MacSpectrum to determine macrophage polarization states [19].

### 2.2. Cytokine Secretion Assay

*C. burnetii* NMII was cultured in acidified citrate cysteine medium-2 (ACCM-2) for 7 days at 37 °C, 5% CO_2_, 2.5% O_2_ [20]. Bacteria were quantified using PicoGreen [21], concentrated by centrifugation (3000× *g*, 10 min, 4 °C), and resuspended in PBS containing 0.25 M sucrose (PBSS) and stored at –80 °C until further use. Before infection, THP-1 cells (American Type Culture Collection, Manassas, VA, USA, TIB-202) were differentiated in RPMI-1640 medium supplemented with 1 mM sodium pyruvate, 0.05 mM beta-mercaptoethanol, 4500 mg/L glucose, and 10% heat-inactivated fetal bovine serum (FBS) at 37 °C under 5% CO_2_ for 24 h using 30 nM phorbol 12-myristate 13-acetate (PMA), followed by 24 h of rest in PMA-free medium. THP-1 macrophages were infected with NMII at an MOI of 25. As positive control for the cytokine assay, THP-1 macrophages were treated with 200 ng/mL of *E. coli* O26:B6 lipopolysaccharide (Sigma-Aldrich, St. Louis, MO, USA) for 3 h and 5 mM ATP (Adenosine 5′-triphosphate disodium salt hydrate; Sigma-Aldrich) for 30 min. Cytokine levels were assessed in cell culture supernatants using a Th1/Th2 Cytokine & Chemokine 20-Plex ProcartaPlex Panel 1 (Invitrogen, Carlsbad, CA, USA) at 48, 72, and 120 hpi according to manufacturer’s guidelines. Briefly, supernatants were centrifuged 10,000× *g* for 10 min to remove particulate matter and stored at −80 °C till further use. In a 96-well plate, magnetic beads were added in appropriate wells, washed, and 50 μL of prepared antigen standards, controls, or samples were added. The plate was shaken for 30 min, 500 rpm at room temperature (RT), followed by overnight incubation at 4 °C. The plate was shaken again for 30 min at RT (500 rpm), washed with 1X Wash Buffer, and incubated with the detection antibody (30 min, RT, 500 rpm). The plate was washed with 1X Wash Buffer, incubated with Streptavidin-Phycoerythrin (SAPE; 30 min, RT, 500 rpm), followed by washing and addition of 120 μL of Reading buffer to analyze median fluorescence intensity (MFI) using a Luminex 200 instrument.

### 2.3. Single-Cell RNA Sequencing

PMA-differentiated THP-1 cells were infected with GFP-tagged NMII (Tn1832) [22] at an MOI of 25 and at 48 hpi fluorescence-activated cell sorting (BD FACSAria Fusion) was carried out to separate infected (GFP-positive) cells from bystander (GFP-negative) cells. Uninfected THP-1 cells were similarly processed to serve as control. We selected 48 hpi for single-cell RNA sequencing (scRNA-seq) because at 72 hpi we were unable to recover sufficient viable cells to meet the minimum threshold of ~1000 cells per condition required for library preparation. scRNA-seq libraries were generated at Yale Center for Genome Analysis from at least 1000 cells from each population by capturing individual cells inside gel beads in emulsion using single cell 3’ v3 chemistry (10X Genomics, Pleasanton, CA, USA). Single-cell sequencing reads were processed for quality control and analyzed to compare cell clustering and differential gene expression within each population using Cell Ranger 3.1 pipeline [23]. Single cell clusters were visualized using tSNE analysis using Loupe Cell Browser (10X Genomics) and log2 fold change was defined as the ratio of UMI (unique molecular identifier) counts in each cluster relative to all other clusters (log2 fold change ≥ 0.75 and *p*-value < 0.05). Differentially regulated genes in each cluster were analyzed using IPA to identify enriched pathways.

## 3. Results

### 3.1. Macrophage Gene Expression Varied Between C. burnetii Infections

**Figure 1:**
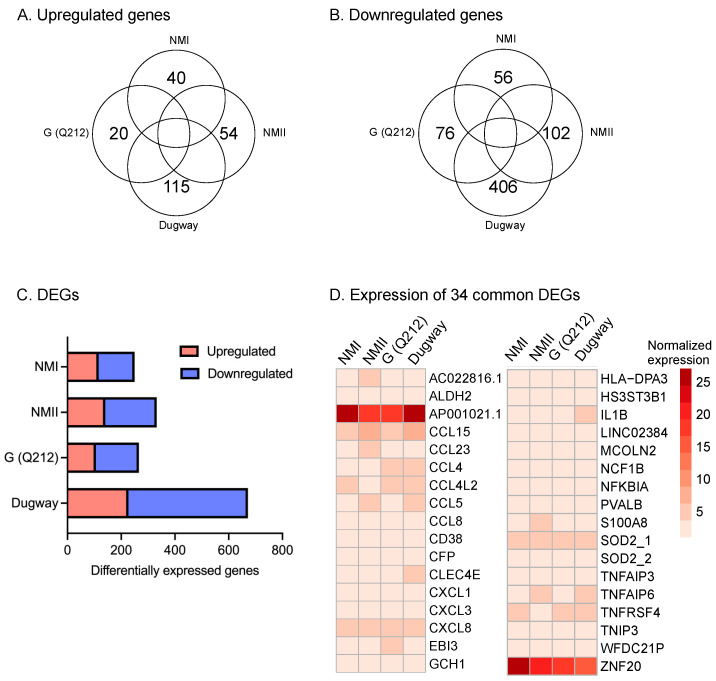
hAMs gene expression in response to *C. burnetii* infection. Venn diagrams showing (**A**) upregulated and (**B**) downregulated genes in primary human alveolar macrophages (hAMs) infected with NMI, NMII, G (Q212), or Dugway isolates of *C. burnetii* compared to uninfected hAMs (−0.75 ≤ log2fc ≥ 0.75, padj ≤ 0.05; *n* = 3) at 72 hpi. (**C**) Distribution of up- and down-regulated genes within differentially expressed genes (DEGs) in hAMs infected with each *C. burnetii* strain. (**D**) Heatmap of DEGs common to all four infections.

We infected hAMs with *C. burnetii* NMI, NMII, G (Q212), or Dugway and measured gene expression at 72 h post-infection (hpi) using RNA sequencing (RNA-seq). This analysis identified hundreds of genes that were differentially expressed in infected macrophages compared to uninfected controls (log2fc ≥ 0.75, padj < 0.05; *n* = 3) (Figure 1, Table S1). Infections with NMI or G (Q212), which are pathogenic to humans, or with NMII, a non-pathogenic strain derived from NMI, resulted in differential expression of roughly the same number of genes: 250, 266, and 332, respectively. In contrast, Dugway, an avirulent strain isolated from rodents, caused the differential expression of 672 genes, with most of the differentially expressed genes (445) showing down-regulation in infected cells compared to uninfected controls. Thirty-four differentially expressed genes (DEGs) were common to all four infections, with majority being inflammation-related genes (Figure 1D).

### 3.2. C. burnetii Activates Macrophage Proinflammatory Pathways

**Figure 2:**
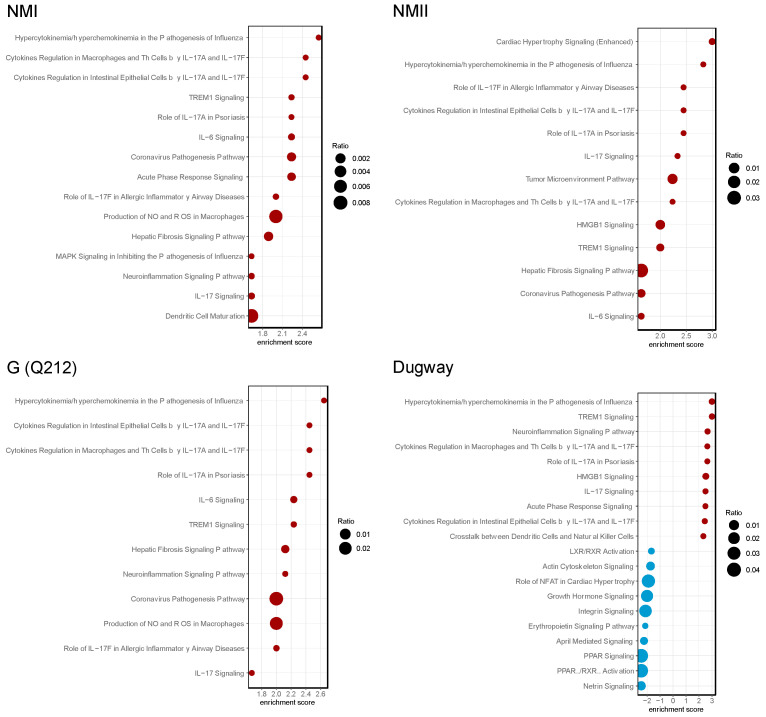
*C. burnetii* strains activate IL-17 signaling pathways. Bubble plots of top 20 enriched pathways in human alveolar macrophages (hAMs) infected with NMI, NMII, G (Q212), or Dugway isolates of *C. burnetii* compared to uninfected hAMs (*n* = 3) at 72 hpi. Red bubbles represent pathways that are activated (z-score ≥ 1.5, *p* < 0.05) and blue bubbles correspond to inhibited pathways (z-score ≤ −1.5, *p* < 0.05). Bubble size corresponds to the ratio of the number of differentially expressed genes in a pathway to the total number of genes in that pathway.

To elucidate the functional implications of differential gene expression, we performed pathway enrichment analysis using the Ingenuity Pathway Analysis (IPA) software package [18]. The IPA data indicated that proinflammatory signaling, including IL-17 signaling, IL-6 signaling and TREM1 signaling, were activated (z-score ≥ 1.5, *p* < 0.05) in hAMs irrespective of the infecting *C. burnetii* isolate (Figure 2, Table S2). IL-17, a member of the IL-17 family of proinflammatory cytokines, induces proinflammatory and regulatory responses in many cell types [24].

**Figure 3:**
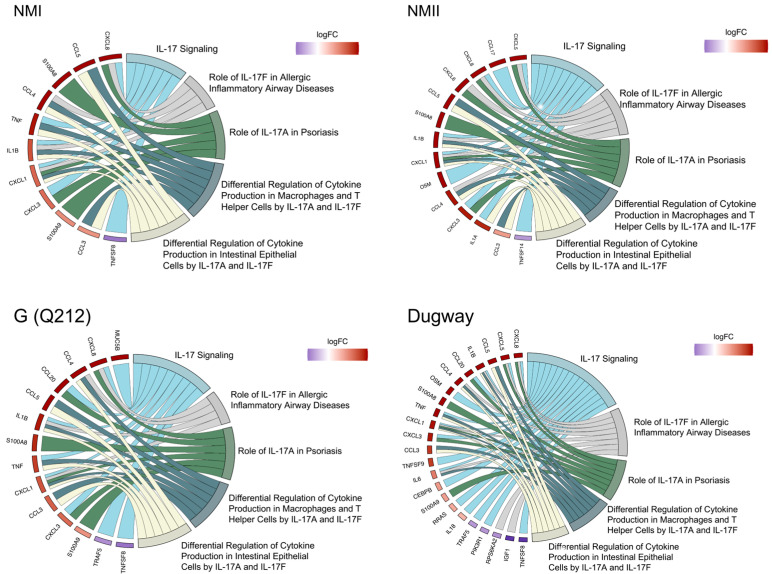
*C. burnetii* strains activate IL-17 signaling. Chord diagrams showing differential expression of genes (**left**) belonging to IL-17 signaling-related pathways (**right**) in human alveolar macrophages (hAMs) infected with NMI, NMII, G (Q212), or Dugway isolates of *C. burnetii* compared to uninfected hAMs (*n* = 3) at 72 hpi.

To identify IL-17 signaling-associated proteins that may participate in *C. burnetii* infection, we used IPA to perform a pathway reconstruction analysis. We found that genes downstream of IL-17 signaling were consistently upregulated in *C. burnetii*-infected hAMs (Figure 3, Table S1). The upregulated genes encode chemokines and cytokines that recruit immune cells or mount proinflammatory host response at the site of intracellular bacterial infection [25,26,27,28,29,30,31].

**Figure 4:**
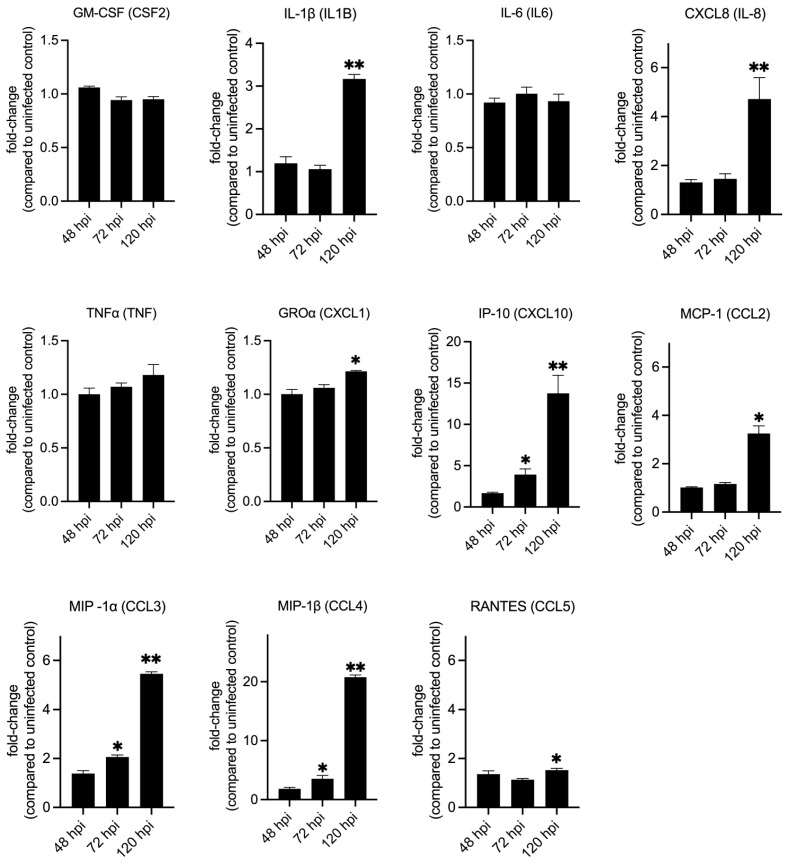
Secretion of cytokines downstream of IL-17 signaling in infected macrophages. Quantification of cytokines and chemokines released from NMII-infected THP-1 macrophages at 48, 72, and 120 h post-infection. Expression values are depicted as fold change between infected over uninfected cells at each timepoint. Statistical significance was calculated using Welch’s t-test with Benjamini–Hochberg FDR correction (* *p*-adj ≤ 0.05, ** *p*-adj ≤ 0.01; *n* = 3).

To validate the gene expression data, we infected THP-1 macrophages with NMII and measured several cytokines that are downstream of IL-17 signaling at 48 h, 72 h, and 120 hpi (Figure 4, Table S3). This assay showed that significantly higher amounts of IL-1β, IL-8, GRO⍶ (CXCL1), CXCL10, CCL2, CCL3, CCL4 and CCL5 were secreted by infected macrophages at 120 hpi. But at 72 hpi only CXCL10, CCL3 and CCL4 were produced at significantly higher amounts in infected cells and at 48 hpi none of the cytokines were significantly different between uninfected and infected cells (Figure 4, Table S3). These data indicate that immune response downstream of IL-17 signaling may not be active in human macrophages during the initial stages of *C. burnetii* infection, as reported previously in mouse macrophages [32,33], but by 120 hpi the proinflammatory IL-17 response is functional.

### 3.3. Macrophage Polarization Varied Between C. burnetii Isolates

**Figure 5:**
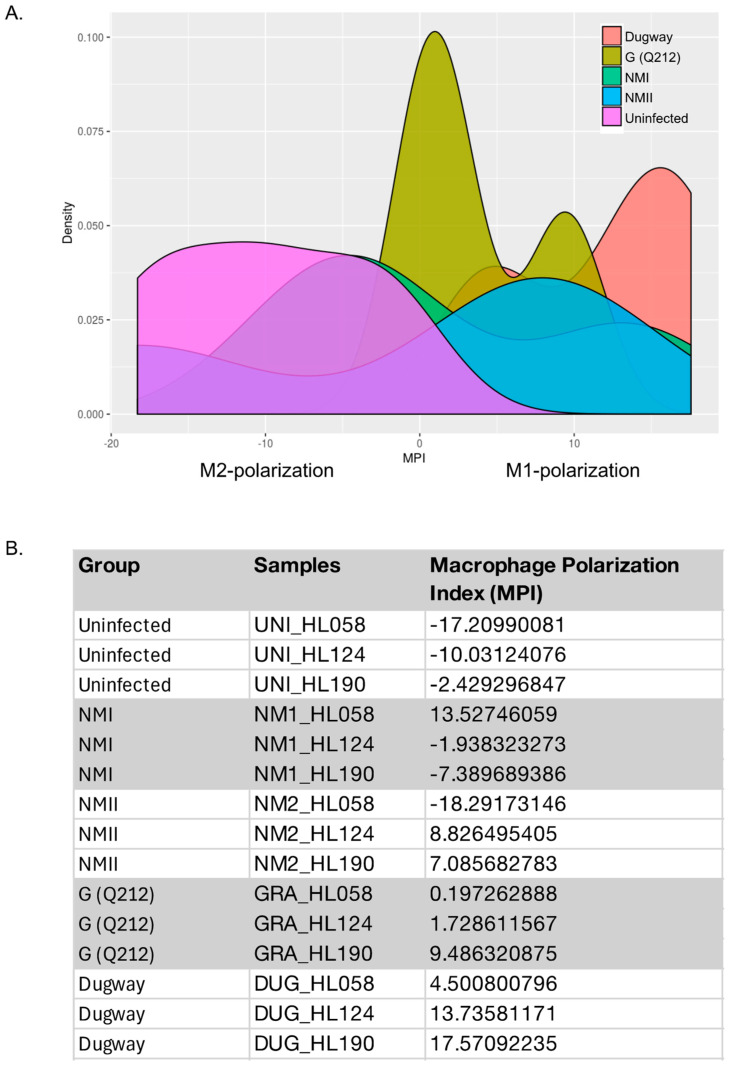
Macrophage polarization. (**A**) Density plots of Macrophage Polarization Index (MPI) for human alveolar macrophages (hAMs) infected with NMI, NMII, G (Q212), or Dugway isolates of *C. burnetii* (72 hpi). MPI were calculated using MacSpectrum and normalized gene expression values. (**B**) Table listing MPI for each sample. hAMs were derived from three donors (HL058, HL124, HL190).

Using MacSpectrum [19], a tool that assigns macrophage polarization index (MPI) based on the expression of a set of genes that are associated with either M1 or M2 activation states, we estimated the polarization states of hAMs infected with NMI, NMII, G (Q212) or Dugway (Figure 5). A high MPI indicates an M1-like (more inflammatory) phenotype, whereas a low MPI suggests an M2-like (less inflammatory) phenotype. The three uninfected samples had low MPI (−17.20, −10.03, −2.43), confirming that the inflammatory pathways in the control cells were not activated. In contrast, the three transcriptomes of Dugway-infected hAMs had high MPI (4.50, 13.74, 17.57), indicating that the rodent isolate likely induced a strong M1-like proinflammatory response in human macrophages. Transcriptional profiles of G (Q212)-infected hAMs also received positive MPI—albeit lower values than Dugway infection—(0.20, 1.73, 9.49). Unlike the MPIs for Dugway and G (Q212) infections, which were relatively consistent across hAMs derived from three donors, infections with NMI and NMII produced a broad range of MPI (NMI: −7.39, −1.94, 13.53; NMII: −18.29, 7.09, 8.83) (Figure 5). This wide distribution of MPI suggests that the immune response to NMI and NMII vary considerably between macrophage populations.

### 3.4. Single-Cell Sequencing Show Heterogenous Macrophage Response to NMII Infection

**Figure 6:**
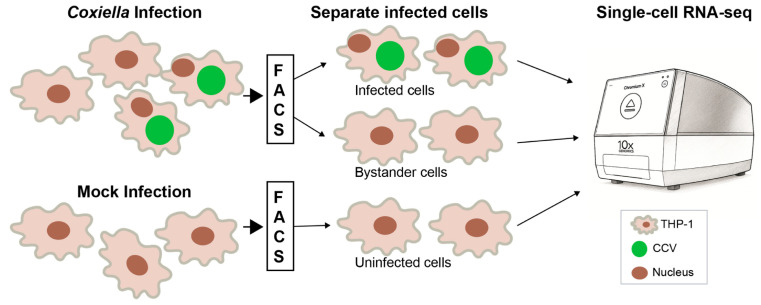
Single-cell RNA-sequencing workflow. THP-1 macrophages infected with GFP-tagged NMII were separated at 48 h post-infection using fluorescence-activated cell sorting (FACS) into infected cells (GFP-positive) and bystander cells (GFP-negative). Mock-infected cells that were passed through the cell sorter were used as controls. Each population was processed using the 10x Genomics Chromium platform for single-cell RNA-sequencing. CCV, *Coxiella*-containing vacuole.

To study macrophage inflammatory response to *C. burnetii* in more detail, we conducted a single-cell RNA-seq (scRNA-seq) analysis. We infected THP-1 macrophages with GFP-tagged NMII [22] and at 48 hpi sorted the cells into GFP-positive (NMII-infected) and GFP-negative (bystander) populations. Uninfected THP-1 macrophages that were processed similarly were used as controls (Figure 6).

**Figure 7:**
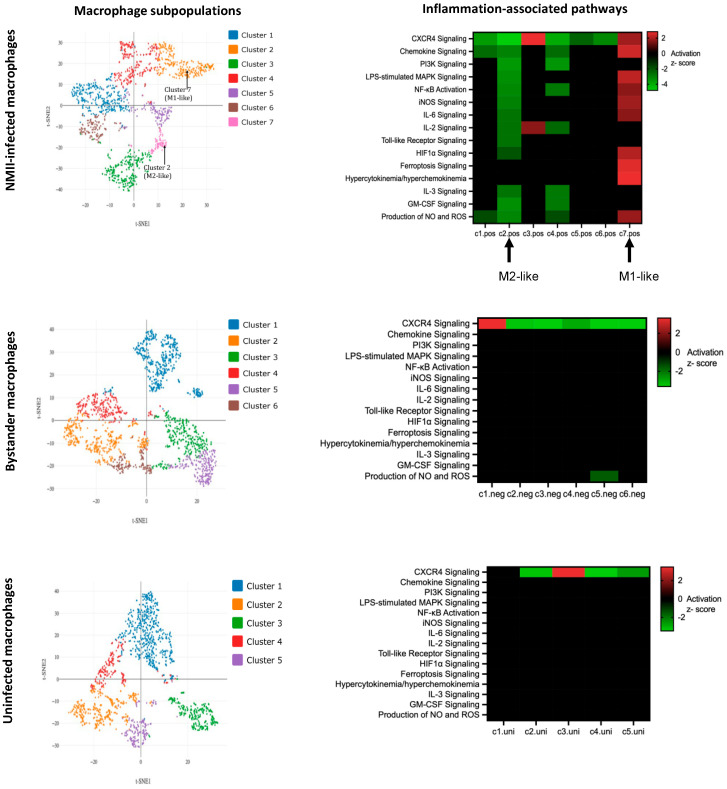
Single-cell analyses show transcriptional heterogeneity. (**Left panels**): Clustering of cells by t-distributed stochastic neighbor embedding (t-SNE) in NMII-infected (GFP-positive), bystander (GFP-negative) and uninfected THP-1 cells. (**Right panels**): Heatmaps depicting enrichment of 15 proinflammatory pathways (y-axes). Clusters within each population is shown on x-axes. Upregulated pathways are in red and downregulated pathways are in green (z-score ≥ 1.5 or ≤−1.5, *p* < 0.05).

Around 1000 cells from each population were subjected to scRNA-seq, which revealed clusters (subpopulations) of cells with differing gene expression patterns within each population (Figure 7). Pathway enrichment analysis of differentially expressed genes in NMII-infected macrophage subpopulations showed significant difference in the activation states of several inflammation-associated pathways (Figure 7, top panel). For example, among 15 inflammation-associated signaling pathways [18,34], 10 proinflammatory pathways, including pathways that are downstream to IL-17 signaling such as IL-6, MAPK, NFKB, iNOS, and NO/ROS signaling were significantly activated (positive z-score) in Cluster 7, whereas the same pathways were inhibited (negative z-score) in Cluster 2. Thus, cells in Cluster 7 appear to show a proinflammatory M1-like macrophage polarization, whereas cells in Cluster 2 have an anti-inflammatory M2-like polarization [35]. Unlike in the NMII-infected macrophages, M1/M2-like polarization was not apparent in the bystander or uninfected populations (Figure 7, middle and bottom panels).

## 4. Discussion

In this study we show that *C. burnetii* likely activates proinflammatory pathways, particularly IL-17 signaling, in human macrophages. However, inflammatory response downstream of IL-17 signaling seemed to be muted during initial stages of *C. burnetii* infection and the magnitude and nature of the inflammatory response varied depending on the bacterial strain and the macrophage subpopulation.

*C. burnetii* Dugway, a rodent-derived avirulent strain, induced a robust transcriptional response in hAMs, including strong activation of proinflammatory pathways and consistent M1-like polarization. In contrast, the human-pathogenic isolates NMI and G (Q212), as well as the attenuated NMII strain, elicited a more subdued and variable response. Dugway infection also seems to cause the downregulation of several signaling pathways, including suppression of PPAR/RXR signaling that could impair lipid metabolism and inhibition of integrin signaling that could disrupt cytoskeletal dynamics. At present it is unknown how, or if, these pathways contribute to the attenuated virulence of Dugway but identifying host processes that reduce *C. burnetii* virulence could contribute to the development of novel host-targeted therapies.

In our data, IL-17 signaling appeared to play a central role in macrophage response to *C. burnetii*. Previous studies have shown that IL-17 signaling provides a protective immune response against other intracellular pathogens such as *L. pneumophila*, *M. tuberculosis*, and *Francisella tularensis* [3,4,36]. IL-17 is a proinflammatory cytokine that activates several independent signaling mechanisms via TRAF (TNF receptor associated factor) and PI3K/AKT signaling [25,37]. These signaling cascades, in turn, lead to the secretion of chemokines and cytokines. Our transcriptomic and cytokine data for IL-17 signaling showed a temporal pattern, with significant gene expression occurring at 72 hpi but significant cytokine secretion occurring only at 120 hpi. In murine alveolar macrophages, *C. burnetii* inhibits IL-17 signaling during early stages of infection via a Type 4 Secretion System-dependent mechanism [32]. Further experiments in human macrophages should clarify whether the lag in cytokine secretion observed in our study is due to a similar process.

Macrophage polarization plays a critical role in shaping the immune response to intracellular pathogens. We observed that Dugway- and G (Q212)-infected hAMs consistently exhibited an M1-like phenotype, characterized by high macrophage polarization index (MPI). In contrast, NMI and NMII infections produced MPI scores that ranged from M2-like to M1-like in hAMs derived from three donors. These data suggest that host genetic or epigenetic factors could influence polarization outcomes, but future studies using macrophages from a larger set of donors will be needed to confirm these results.

Variability was also observed in the scRNA-seq data, which revealed distinct macrophage subpopulations with divergent polarization states in NMII-infected THP-1 macrophages. These findings underscore the importance of cellular heterogeneity in host–pathogen interactions and highlight the limitations of bulk transcriptomic analyses, which may obscure critical subpopulation dynamics. scRNA-seq also allowed us to compare gene expression in bystander cells to that in infected and uninfected cells. While the patterns of gene expression in bystander and uninfected populations were very similar, infected macrophages showed increased expression of genes linked to lipid metabolism, iron homeostasis and oxidative stress defense compared to bystander cells. Elevated expression of lipid-associated genes such as APOE, APOC1, PSAP, and ASAH1 indicates remodeling of lipid transport and lysosomal metabolism, processes that *C. burnetii* exploits for vacuole formation [7,8]. Upregulation of FTH1 and FTL suggests enhanced sequestration of iron, likely limiting its availability to *C. burnetii*, and elevated PRDX1 expression points to strengthened antioxidant capacity. Future functional studies will be needed to confirm these observations and to clarify the roles of these host responses during *C. burnetii* infection.

Besides the induction of proinflammatory signaling, our gene expression analyses identified the activation of several pro-survival pathways, including PI3K/Akt signaling, autophagy, toll-like receptor signaling, TGF-β signaling, JAK/STAT signaling, STAT3 pathway, and MAPK signaling in *C. burnetii* infected hAMs. Our data also revealed putative roles for Wnt/Ca+ pathway and ferroptosis signaling that have not previously been described in the context of *C. burnetii* infection but are involved in infections with other pathogens such as *Ehrlichia chaffeensis* and *M. tuberculosis* [38,39]. Going forward, pursuing functional investigations of these pathways would likely advance our understanding of how human macrophages respond to *C. burnetii* infection.

In summary, our findings highlight the complexity of macrophage responses to *C. burnetii* and underscore the importance of strain-specific and cell-specific factors in shaping host immunity. However, this study has several limitations that should be considered when interpreting the results. Bulk transcriptomic profiling was performed in primary human alveolar macrophages (hAMs), whereas cytokine assay and scRNA-seq were limited to THP-1 macrophages and only with the NMII strain, which may restrict physiological relevance. THP-1 and NMII were used for validation experiments due to practical and biosafety considerations: THP-1 cells provide a reproducible human macrophage model and NMII strain allows experiments to be conducted under less restrictive biosafety level 2 conditions. The small number of human lung donors (*n* = 3) and inter-individual variability further limit the generalizability of the transcriptomic data from hAMs. Additionally, bulk RNA-seq was conducted at 72 hpi, potentially overlooking early and late transcriptional dynamics, and scRNA-seq was performed at 48 hpi, complicating temporal comparisons between bulk and single-cell RNA-seq results. The scRNA-seq dataset also included a relatively low number of cells (~1000 per condition) and lacked biological replicates, reducing statistical power. Finally, the absence of functional experiments, such as IL-17 blockade or manipulation of the Type IV secretion system, precludes definitive conclusions about causality in the observed signaling and polarization patterns. Future studies addressing these limitations will be critical for a more comprehensive understanding of host–*C. burnetii* interactions.

## Data Availability

The data presented in this study are openly available in NCBI Sequence Read Archive (SRA) at BioProject accession PRJNA679931.

## Supplementary Materials

The following supporting information can be downloaded at https://www.mdpi.com/article/10.3390/pathogens14111101/s1, Table S1: Differentially expressed genes in hAMs infected with *C. burnetii* strains.; Table S2: Enriched pathways in hAMs infected with *C. burnetii* strains; Table S3: Cytokine secretion assay.
